# Ageism: Does it Exist Among Children?

**DOI:** 10.1100/tsw.2007.171

**Published:** 2007-07-27

**Authors:** Mladen Davidovic, Zorana Djordjevic, Predrag Erceg, Nebojsa Despotovic, Dragoslav P. Milosevic

**Affiliations:** Gerontology Clinic, “Zvezdara” University Hospital, Dimitrija Tucovica 161, 11000 Belgrade, Serbia

**Keywords:** ageism, aging, elderly, children, stereotyping, discrimination

## Abstract

Ageism is stereotyping and prejudice against individuals or groups because of their age. Robert Butler first used it in 1969, to express a systematic stereotyping and discrimination against elderly people. Available data appears to confirm that attitudes of children to the old age differ from that of adults. The study population consisted of 162 subjects (56 school children, 48 nurses and 58 elderly patients). Each subject in the survey was asked to respond to the following three questions: Question #1: “Is the old age unattractive ?”; Question #2: “How old is an old man? Question #3: “What should you do to have a long life (what is good for longevity)? The majority of polled children (33) gave positive statements about ageing in their responses to the first item, while most of the nurses gave condition answers, like: “It is not unattractive if you are healthy”. Elderly subjects made up a group with the majority of negative responses (in percentage), as only 33% of them answered that old age is not unattractive. All three groups of subjects demonstrated a good knowledge of what is considered good for longevity, and had a generally positive health attitude. Our results indicate that majority of children have positive perception and attitude about old age, which leads us to conclusion that ageism is adopted later in life.

## INTRODUCTION

The term "ageism" was used for the first time in 1969 by Robert Butler to express in one word a process of systematic stereotyping and discrimination against people on the grounds of their old age[1]. Research has shown that ageism is prevalent in today's society and widespread in all social structures and among different age groups[2]. The destructive impact of ageism is visible in three major areas: discrimination in the workplace, bias in the healthcare system, and social prejudice[3,4,5].

Recent investigations on ageism have found that older individuals are generally perceived less favorable than those who are younger[6,7]. Younger individuals and males scored significantly higher on the Fraboni Scale of Ageism than older individuals and women[8].

Previous studies on attitudes of children towards elderly have shown contradictory results. While most of the papers reported that children's perception of older adults tended to be negative, there was a number of studies that showed no significant differences in attitudes between young and old[9,10]. Some of these studies reported positive perception of older people[11,12,13,14]. Possible explanation of these contrasting findings could be in different methodology used for assessing stereotypes about older persons[11,12].

In order to examine perceptions and attitudes of children about elderly people, and to probe attitudes that nurses and elderly patients have towards the old age, we ran a survey in three population groups: school children, nurses and elderly patients. Furthermore, the study wanted to test what the groups knew about healthy aging and longevity.

## METHODS

The study population consisted of 162 subjects (56 school children, 48 nurses and 58 elderly patients). School children were recruited from outpatient pediatric clinic, and they were not seriously ill. Their age range was from 10 to 16, with a mean age of 13 years. Their previous experience with aging originated exclusively from family members (grandparents). Nurses were recruited from Gerontology Clinic, and their age range was from 20 to 47, with a mean age of 34 years. All were qualified nurses with completed nursing school and had a working experience at the geriatric ward. Elderly patients were recruited from Gerontology Clinic, and their age range was from 65 to 85, with a mean age of 75 years. All elderly subjects were hospitalized in geriatric ward for diagnosis and treatment of chronic illnesses. Patients suffering from dementia were excluded from the study. Subjects' educational level varied from primary school to university level.

The survey was conducted in the form of questionnaire, consisting of three simplified questions, presented in a way that is understandable for all subject groups, regardless to their age, education level and social status.

The questions were as follows:

Question #1: "Is an old age unattractive?"
Question #2: "How old is an old man?"
Question #3: "What should you do to live a long life (what is good for longevity)?

The questionnaire did not contain predefined answers. Subjects were instructed to provide answers in their own words and give a short explanation.

## RESULTS

### Responses of Children

The majority of children – 33 out of 56 (59%) – gave a negative response to question "Is an old age unattractive?" which means that they had a positive attitude towards old age and aging. Sixteen children (29%) said the old age was unattractive, while seven (12%) of those polled expressed opinion that growing old was not that bad. When asked to explain their attitude, the vast majority of children who responded that an old age was unattractive said: "Old age is unattractive because you are closer to death."

All children answered the question "How old is an old man?" in numbers representing a certain age. Their responses ranged from 35-80 years, with mean value of 63 years.

Almost all school-age subjects had a very good knowledge of what is considered as healthy life as one of preconditions for longevity. Asked to say "What should you do to live long life (what is good for longevity)?" 54 (96%) of subjects responded that one should practice a healthy diet (rich in vegetables, fruit, cereals, small quantities of meat) and physical activity.

## Responses of Nurses

The majority of nurses, 41 out of 48 (85%), gave a conditional response to the question "Is an old age unattractive?" ("It is not unattractive if you are healthy" or "Depends on a person and his/hers attitude"). Four nurses (8%) said that old age was not unattractive, while three respondents (7%) had the opposite opinion.

Asked to say "How old is an old man?" the nurses provided answers that ranged from 50-75 with mean value of 60 years. A number of nurses remarked that age of the old person depended on how that person felt towards her own age.

The nurses demonstrated an excellent knowledge of healthy lifestyles. All responders gave a correct answer to the question "What should you do to live long life (what is good for longevity)?

## Responses of Elderly

The majority of elderly patients displayed a negative attitude towards aging and old age. Thirty-nine of 58 respondents (67%) gave positive answer to a question "Is an old age unattractive?" while the majority of the remaining 33% responders who gave negative responses, remarked that an old age is not unattractive if one is in good health.

Asked to say "How old is an old man?" only 54 % of the responders expressed themselves in numbers. Their responses ranged from 45 to 80 years, with mean value of 67.

Elderly patients demonstrated a good knowledge of preconditions for longevity, as 98% of them gave correct answers to the question "What should you do to live long life (what is good for longevity)?

Responses to questions are shown in Table 1.

**Table 1**
**Subject's Responses to the Questionnaire**

| | ***Children's Responses n=56*** | ***Nurses Responses n=48*** | ***Elderly Responses n=58*** |
|---|---|---|---|
| Question #1: "Is an old age unattractive?" | No 33 (59%)<br>Yes 16 (29%)<br>Not so depressing 7 (12%) | No 4 (8%)<br>Conditional (ie. No, if the person is healthy) 41 (85%)<br>Yes 3 (7%) | No 19 (33%)<br>Yes 39 (67%) |
| Question #2: "How old is an old man?" | 35-80 years (mean 63) | 50-75 years (mean 60) | 45-80 (mean 67) |
| Question #3: "What should you do to live long life (what is good for longevity)? | Correct answer 54 (96%)<br>Wrong answer 1 (2%)<br>No answer 1 (2%) | Correct answer 48 (100%)<br>Wrong answer 0 (0%) | Correct answer 57 (98%)<br>Wrong answer 1 (2%) |

## DISCUSSION

## Responses of Children

After processing the data from our questionnaire, we were surprised by a high percentage of children who did not consider an old age as "unattractive." The majority of previous studies have shown that children usually have negative perception of aging and old age[7,9,10,15,16,17,18], though there were other works that presented positive or neutral attitudes of children towards the old people[11,12,13,14]. Different methodology and usage of questionnaires unsuitable for children contributed to different and contradictory findings in previous studies. Using the Children's Views on Aging (CVoA) questionnaire, Marks et al. found that, although children's attitudes toward the aging process were often negative, their general attitudes about older adults were positive[13]. Using the same questionnaire, Newman et al. found that children did not have negative perception and attitude towards ageing and old age[11]. They have also demonstrated that daily contact between children and old people was of quintessential importance for breaking the prejudices. Having tested the schoolchildren who took part in an inter-generational program, before and after having the old persons present in their classes, they found that attitudes of children towards the old people were more positive in the second test, while a certain number of previously indecisive responders changed their position to positive attitude[11]. These findings encourage having more inter-generational programs, which, we believe, are the best way to counter the possible negative attitudes and prejudices to old people and aging. Previous researches demonstrated the benefit of inter-generational programs both for children and for elderly[19,20].

Asked to provide more detailed explanation for their negative attitudes towards aging, the majority of children gave answers that could be summarized as follows: “Old age is unattractive because you are closer to death.” Explanation for this lies in the fact that certain children have, in fact, a negative attitude towards the process of ageing, and not towards the old people, which is in accordance with previous findings by marks et al.[13].

In our study, the school children have shown an excellent knowledge of the aging process. When asked “how old is an old man”, they all gave a precise number as an answer. The mean value of their answers was 63 years, a figure very close to a well-known definition. A similar response was given by college students in a previous study (61 year)[21]. Furthermore, almost al respondents knew very well what was considered good for longevity, as they all cited healthy diet and regular physical exercise.

## Responses of Nurses

The majority of nurses gave a conditional response to the question “Is an old age unattractive?” indicating that they did not have negative attitude towards old age. Only a small number of them found old age unattractive, an unexpected result, bearing in mind that geriatric nurses are dealing with suffering and dying of an elderly patients every day. However, a number of them remarked that old age was not unattractive if a person is in good health. Our results are different from findings presented in majority of the studies that reported negative attitudes which nurses hold towards older people[22,23]. Training, rewards and less workload on the nurses is needed to improve the situation in the field[24,25].

All nurses demonstrated an excellent knowledge about aging process and had a positive health attitude, which was expected from them as health professionals.

## Responses of Elderly

The majority of elderly patients displayed a negative attitude towards aging and old age. This could be due to the fact that our elderly subjects were suffering from chronic diseases and were hospitalized at the geriatric ward. This finding is opposite to results reported by Rupp et al. who found that older people had no prejudices against their own group[8].

Our subjects had positive health attitude and good knowledge about aging, which was encouraging.

## Future Implications

As more and more people live longer, and the number of older adults increases, it is important for society to discover the nature and genesis of the attitudes and perceptions of its younger population toward older adults[11]. Our results indicate that majority of children have positive perception and attitude about old age, which leads us to conclusion that ageism is adopted later in life. This finding should encourage us to develop strategies to prevent formation of prejudices against elderly. Most authors agree that educational and intergenerational programs are the best way to achieve this[26,27,28,29,30].

## CONCLUSIONS

Our results indicate that majority of children have positive perception and attitude about an old age, which leads us to conclusion that ageism is adopted later in life. This finding should encourage us to develop strategies to prevent formation of prejudices against elderly.
